# Sonogashira Reaction of Aryl and Heteroaryl Halides with Terminal Alkynes Catalyzed by a Highly Efficient and Recyclable Nanosized MCM-41 Anchored Palladium Bipyridyl Complex

**DOI:** 10.3390/molecules15129157

**Published:** 2010-12-10

**Authors:** Bo-Nan Lin, Shao-Hsien Huang, Wei-Yi Wu, Chung-Yuan Mou, Fu-Yu Tsai

**Affiliations:** 1 Institute of Organic and Polymeric Materials, National Taipei University of Technology, Taipei 106, Taiwan; 2 Department of Chemistry, National Taiwan University, Taipei 106, Taiwan

**Keywords:** Sonogashira reaction, mesoporous silica, palladium complex, recyclable catalyst, heterogeneous catalysis

## Abstract

A heterogeneous catalyst, nanosized MCM-41-Pd, was used to catalyze the Sonogashira coupling of aryl and heteroaryl halides with terminal alkynes in the presence of CuI and triphenylphosphine. The coupling products were obtained in high yields using low Pd loadings to 0.01 mol%, and the nanosized MCM-41-Pd catalyst was recovered by centrifugation of the reaction solution and re-used in further runs without significant loss of reactivity.

## 1. Introduction

The reaction of aryl halides or vinyl halides with terminal alkynes catalyzed by a Pd(II)/Cu(I) system is known as the Sonogashira coupling, and is one of the most powerful methods for the straightforward construction of *sp*^2^–*sp* carbon–carbon bonds in synthetic chemistry [1,2,3,4,5,6,7]. This methodology has been widely applied to prepare biologically-active molecules [8,9,10,11,12,13], natural products [14,15,16,17], conducting polymers/engineering materials [18,19], and macrocycles with acetylene links [20,21].

The Sonogashira reaction is, in general, carried out in a homogeneous phase [22], and therefore the recovery of expensive palladium complexes, facile separation of catalysts and products, and industrial application are major aims for the benefit of both economy and the environment. For these reasons, heterogenization of the homogeneous Sonogashira reaction has become an aim of great interest to chemists in recent years. Choudary and co-workers described a layered double hydroxide-supported nanopalladium catalyst for the coupling of aryl chlorides and phenylacetylene [23], and Pd/C has been used to catalyze the Sonagashira reaction of aryl halides with acetylenes [24,25,26,27,28,29,30], while PVP-supported nanoparticle palladium metal can be employed for the coupling of aryl iodides and bromides with terminal alkynes [31]. Djakovitch and co-workers reported that microporous [Pd-Cu]/NaY [32], [Pd(NH_3_)_4_]^2+^/(NH_4_)Y [33], and [Pd(NH_3_)_4_]^2+^/NaY [34] systems can be applied in the Sonogashira reaction using 1–2 mol% of the Pd catalyst, and palladium can be also supported by silica in order to create a recyclable catalyst for use in the Sonogashira reaction [35].

**Figure 1:**
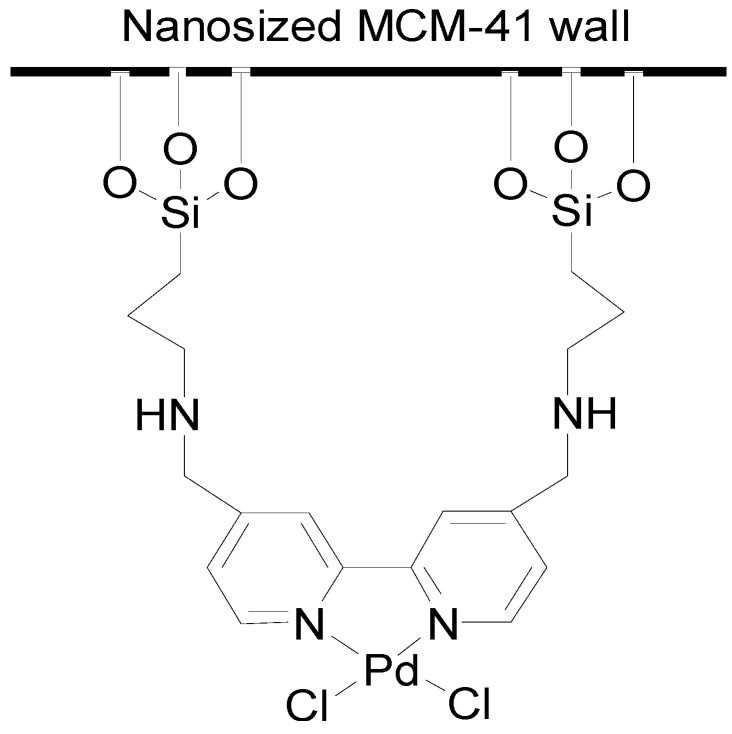
NS-MCM-41-Pd.

Mesoporous silica is becoming more and more widely used as a solid support owing to its well-defined structure, uniform pore size, high surface area, and large number of silanol groups for the grafting of metal complexes [36,37,38,39,40,41,42,43,44]. Djakovitch’s group prepared a mesoporous [Pd]/SBA-15 catalyst to demonstrate that larger aryl halides such as bromoanthacene can be active in this catalytic system, whereas the microporous support [Pd(NH_3_)_4_]^2+^/NaY is inactive [45]. Cai and co-workers employed MCM-41-supported sulfur palladium [46], bidentate phosphine palladium [47], and thioether palladium [48] systems to catalyze the coupling of aryl iodides and terminal alkynes after reduction of the catalyst. Although most known heterogeneous catalysts have been demonstrated to be able to be recycled for use in further runs, the use of catalytic amounts of 0.2–5 mol% of Pd for the Sonagashira reaction is still too high for a single batch reaction when compared with homogeneous catalysts [49,50,51,52,53,54]. We have recently prepared a nanosized MCM-41 grafted palladium bipyridyl complex, NS-MCM-41-Pd (Figure 1), as a highly efficient and recyclable catalyst for the Mizoroki-Heck reaction [55], Kumada-Tamao-Corriu reaction [56], ketone formation [57], and ynone formation [58], which require a very low catalyst loading for a single batch reaction. 

**Scheme 1:**
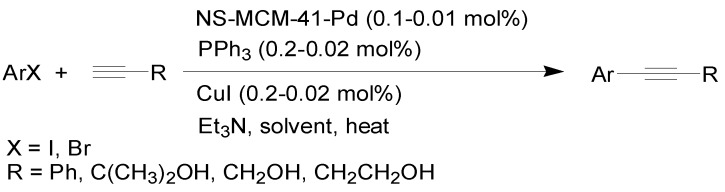
NS-MCM-41-Pd-catalyzed Sonogashira reaction.

The major advantage of this catalyst is that the short and highly-connective wormhole-like channels of nanosized MCM-41 lead to the easy exchange of reactants, salts and products throughout the nanochannels, avoiding saturation of activity. In this paper, we report the use of nanosized MCM-41-Pd to catalyze the coupling of aryl and heteroaryl halides with phenylacetylene and alkynols with high efficiency under Sonogashira reaction conditions using a catalyst loading as low as 0.01 mol%, with the ability to recycle the catalyst for further use (Scheme 1).

## 2. Results and Discussion

### 2.1. Optimization of reaction conditions for the Sonogashira reaction catalyzed by NS-MCM-41-Pd

The procedure for the synthesis of the catalyst, NS-MCM-41-Pd, was presented in our previous reports. After the grafting of the palladium bipyridyl complex onto NS-MCM-41, the surface area and pore diameter decreased from 705 m^2^/g and 2.5 nm to 588 m^2^/g and 2.3 nm, respectively, and the amount of Pd complex anchored to the wall of NS-MCM-41 was quantified to be 0.15 mmol/g by ICP-MASS analysis. In order to optimize the conditions for this prepared heterogeneous catalyst, the solvent effect was first examined using iodobenzene (**1a**) and phenylacetylene (**2a**) as representative reactants. Reactions were carried out in the presence of 0.1 mol% catalyst, 0.2 mol% CuI, 0.2 mol% PPh_3_, and Et_3_N at 50 °C under N_2_ for 3 h. The results are summarized in Table 1, and it was found that Et_3_N was the best solvent for this reaction (Table 1, Entry 1). 

**Table 1:** NS-MCM-41-Pd-catalyzed Sonogashira coupling reaction of iodobenzene **1a** with phenylacetylene **2a**.^a^

Entry	Pd (mol%)	CuI (mol%)	PPh_3_ (mol%)	Solvent	Base	Yield (%)^b^
1	0.1	0.2	0.2	Et_3_N	Et_3_N	97
2	0.1	0.2	0.2	Toluene	Et_3_N^c^	60
3	0.1	0.2	0.2	DMF	Et_3_N^c^	34
4	0.1	0.2	0.2	DMSO	Et_3_N^c^	26
5	0.1	0.2	0.2	NMP	Et_3_N^c^	5
6	0.1	0.2	0	Et_3_N	Et_3_N	38
7	0.1	0	0.2	Et_3_N	Et_3_N	0
8	0.1	0	0	Et_3_N	Et_3_N	0
9	0.1	0.2	0.2	Toluene	KOH^c^	0
10	0.1	0.2	0.2	DMF	KOH^c^	0
11	0.1	0.2	0.2	Toluene	K_2_CO_3_^c^	0
12	0.1	0.2	0.2	DMF	K_2_CO_3_^c^	0

^a ^Reaction conditions: [**1a**]:[**2a**]:[Pd] = 1000:1100:1, at 50 °C for 3 h. ^b ^Isolated yields. ^c ^3 equiv based on **1a** was used as a base.

The use of toluene, DMF, DMSO, and NMP as solvents resulting in lower rates of conversion (Entries 2–5). In the absence of triphenylphosphine, this Sonogashira coupling reaction still proceeded, but with less satisfactory yields (Entry 6). However, the co-catalyst CuI appeared to be necessary for the coupling reaction (Entries 7–8). Regarding the use of a base, inorganic bases such as KOH and K_2_CO_3_ were also examined, but under these conditions the desired product was not obtained due to the poor solubility of these salts in organic solvents (Entries 9–12) [55].

### 2.2. Sonogashira reaction of aryl halides with phenylacetylene

Following optimization of the reaction conditions, the reactions of **2a** with various aryl halides were screened in the subsequent investigation (Table 2). 

**Table 2:** Sonogashira reaction of aryl halides (**1**) with phenylacetylene (**2a**) catalyzed by nanosized MCM-41-Pd.^a^

Entry	Aryl halide		Pd (mol%)	Solvent/Base	T (°C)	t (h)	Yield (%)^b^	TON
1	C_6_H_5_I	**1a**	0.1	Et_3_N/Et_3_N	50	3	**3a**, 97	970
2	C_6_H_5_I	**1a**	0.01	Et_3_N/Et_3_N	50	12	**3a**, 98	9800
3	4-IC_6_H_4_CN	**1b**	0.1	Et_3_N/Et_3_N	50	3	**3b**, 96	960
4	4-IC_6_H_4_CN	**1b**	0.01	Et_3_N/Et_3_N	50	9	**3b**, 96	9600
5	4-MeOC_6_H_4_I	**1c**	0.1	Et_3_N/Et_3_N	50	24	**3c**, 87	870
6	C_6_H_5_Br	**1d**	0.1	NMP/Bu_3_N^c^	140	24	**3a**, 30	300
7	C_6_H_5_Br	**1d**	0.1	Toluene/Bu_3_N^c^	100	24	**3a**, 56	560
8	4-BrC_6_H_4_CN	**1e**	0.1	Et_3_N/Et_3_N	90	3	**3b**, 93	930
9	4-MeCOC_6_H_4_Br	**1f**	0.1	NMP/Et_3_N^c^	90	6	**3d**, 98	980
10	4-NO_2_C_6_H_4_Br	**1g**	0.01	NMP/Et_3_N^c^	90	6	**3e**, 99	9900
11	4-ClC_6_H_4_Br	**1h**	0.1	NMP/Et_3_N^c^	90	24	**3f**, 46	460
12	4-MeOC_6_H_4_Br	**1i**	0.1	NMP/Et_3_N^c^	90	72	**3c**, 40	400
13	2-Bromothiophene	**1j**	0.1	NMP/Et_3_N^c^	90	48	**3g**, 71	710
14	3-Bromothiophene	**1k**	0.1	NMP/Et_3_N^c^	90	96	**3h**, 36	360
15	2-Bromopyridine	**1l**	0.1	NMP/Et_3_N^c^	90	3	**3i**, 99	990
16	3-Bromopyridine	**1m**	0.1	NMP/Et_3_N^c^	90	24	**3j**, 98	980

^a ^Reaction conditions: [**1**]:[**2a**] = 1:1.1, [Pd]:[CuI]:[PPh_3_] = 1:2:2. ^b ^Isolated yields. ^c ^3 equiv based on **1** was used as a base.

Reactions of **2a** with aryl iodides proceeded well with the use of 0.1 mol% NS-MCM-41-Pd at 50 °C (Table 2, Entries 1, 3, and 5), and it should be noted that the same good yields were also obtained in these reactions when using a lower amount of catalyst (0.01 mol%) (Entries 2 and 4). However, the Sonogashira reaction of bromobenzene (**1d**) and **2a** under the same conditions did not afford any product, but replacing the Et_3_N solvent by NMP, a typical solvent for such coupling reactions, resulted in the formation of **3a** in a 30% yield at an elevated temperature (Entry 6), and a yield of up to 56% could be achieved by performing the reaction in toluene at 100 °C for 24 h (Entry 7). Using activated aryl bromides such as 4-bromobenzonitrile (**1e**), 4-bromoacetophenone (**1f**), and 4-bromonitrobenzene (**1g**), better yields of the coupling reactions were observed (Entries 8–10). In the case of the coupling of **1h** with **2a**, the C–Cl bond was inert under the reaction conditions, while the product coupled through the C–Br bond was obtained in a 46% yield (Entry 11). Next, we studied the coupling of halothiophenes and halopyridines with **2a**, and it appeared that 2-bromothiophene (**1j**) and 2-bromo-pyridine (**1l**) resulted in better yields than the corresponding bromides at the 3-position (Entries 13–16).

### 2.3. Sonogashira reaction of aryl halides with alkynols

Under similar conditions, NS-MCM-41-Pd-catalyzed Sonogashira coupling of a wide variety of aryl halides with 2-methyl-3-butyn-2-ol (**4a**) was also achieved (Table 3), and a reaction temperature of 90 °C was found to be optimal. Aryl iodides reacted with **4a** to give the corresponding coupling products in good to excellent yields (Entries 1–3), whereas the use of deactivated bromides as substrates resulted in lower yields (Entry 4). Reactions of activated bromides delivered better conversion rates (Entries 5–9): for example, the catalyst had a turnover number (TON) of 9,600 for the coupling of **1e** with **4a** (Entry 7), and for the heteroaryl halides (Entries 11–15), the catalyst exhibited great activity, with the exception of the reactions of **1k** (Entries 12).

**Table 3:** Sonogashira reaction of aryl halides **1** with alkynols **4** catalyzed by nanosized MCM-41-Pd.^a^

Entry	Aryl halide		Alkynyl alcohol		Pd (mol%)	*t* (h)	Yield (%)^b^	TON
1	C_6_H_5_I	**1a**	HC≡CC(CH_3_)_2_OH	**4a**	0.1	3	**5a**, 94	940
2	4-IC_6_H_4_CN	**1b**	HC≡CC(CH_3_)_2_OH	**4a**	0.1	3	**5b**, 98	980
3	4-MeOC_6_H_4_I	**1c**	HC≡CC(CH_3_)_2_OH	**4a**	0.1	72	**5c**, 61	610
4	C_6_H_5_Br	**1d**	HC≡CC(CH_3_)_2_OH	**4a**	0.1	96	**5a**, 21	210
5	4-BrC_6_H_4_CN	**1e**	HC≡CC(CH_3_)_2_OH	**4a**	0.1	3	**5b**, 98	980
6	4-BrC_6_H_4_CN	**1e**	HC≡CC(CH_3_)_2_OH	**4a**	0.01	12	**5b**, 96	9600
7	4-MeCOC_6_H_4_Br	**1f**	HC≡CC(CH_3_)_2_OH	**4a**	0.1	3	**5d**, 98	980
8	4-NO_2_C_6_H_4_Br	**1g**	HC≡CC(CH_3_)_2_OH	**4a**	0.1	3	**5e**, 97	970
9	4-ClC_6_H_4_Br	**1h**	HC≡CC(CH_3_)_2_OH	**4a**	0.1	24	**5f**, 69	690
10	4-MeOC_6_H_4_Br	**1i**	HC≡CC(CH_3_)_2_OH	**4a**	0.1	96	**5c**, 20	200
11	2-Bromothiophene	**1j**	HC≡CC(CH_3_)_2_OH	**4a**	0.1	48	**5g**, 99	990
12	3-Bromothiophene	**1k**	HC≡CC(CH_3_)_2_OH	**4a**	0.1	96	**5h**, 59	590
13	2-Bromopyridine	**1l**	HC≡CC(CH_3_)_2_OH	**4a**	0.1	3	**5i**, 99	990
14	3-Bromopyridine	**1m**	HC≡CC(CH_3_)_2_OH	**4a**	0.1	6	**5j**, 98	980
15	3-Bromopyridine	**1m**	HC≡CC(CH_3_)_2_OH	**4a**	0.01	24	**5j**, 34	3400
16	C_6_H_5_I	**1a**	HC≡CCH_2_OH	**4b**	0.1	12	**5k**, 85	850
17	C_6_H_5_I	**1a**	HC≡CCH_2_OH	**4b**	0.01	24	**5k**, 84	8400
18	4-IC_6_H_4_CN	**1b**	HC≡CCH_2_OH	**4b**	0.1	3	**5l**, 83	830
19	C_6_H_5_Br	**1d**	HC≡CCH_2_OH	**4b**	0.1	96	**5k**, 10	100
20	4-BrC_6_H_4_CN	**1e**	HC≡CCH_2_OH	**4b**	0.1	24	**5l**, 71	710
21	4-BrC_6_H_4_COMe	**1f**	HC≡CCH_2_OH	**4b**	0.1	3	**5m**, 98	980
22	4-BrC_6_H_4_COMe	**1f**	HC≡CCH_2_OH	**4b**	0.01	48	**5m**, 98	9800
23	4-BrC_6_H_4_NO_2_	**1g**	HC≡CCH_2_OH	**4b**	0.1	24	**5n**, 99	990
24	4-BrC_6_H_4_Cl	**1h**	HC≡CCH_2_OH	**4b**	0.1	72	**5o**, 15	150
25	2-Bromothiophene	**1j**	HC≡CCH_2_OH	**4b**	0.1	48	**5p**, 18	180
26	3-Bromothiophene	**1k**	HC≡CCH_2_OH	**4b**	0.1	96	**5q**, 8	80
27	2-Bromopyridine	**1l**	HC≡CCH_2_OH	**4b**	0.1	3	**5r**, 81	810
28	3-Bromopyridine	**1m**	HC≡CCH_2_OH	**4b**	0.1	48	**5s**, 65	650
29	C_6_H_5_I	**1a**	HC≡CCH_2_CH_2_OH	**4c**	0.1	6	**5t**, 78	780
30	4-MeOC_6_H_4_I	**1c**	HC≡CCH_2_CH_2_OH	**4c**	0.1	12	**5u**, 45	450
31	4-BrC_6_H_4_CN	**1e**	HC≡CCH_2_CH_2_OH	**4c**	0.1	12	**5v**, 99	990
32	4-BrC_6_H_4_COMe	**1f**	HC≡CCH_2_CH_2_OH	**4c**	0.1	12	**5w**, 89	890
33	2-Bromothiophene	**1j**	HC≡CCH_2_CH_2_OH	**4c**	0.1	12	**5x**, 32	320
34	2-Bromopyridine	**1l**	HC≡CCH_2_CH_2_OH	**4c**	0.1	12	**5y**, 64	640

^a ^Reaction conditions: [**1**]:[**4a** or **4c**] = 1:1.1; [**1**]:[**4b**] = 1:1.5; [Pd]:[CuI]:[PPh_3_] = 1:2:2; Et_3_N was used as the solvent and base at 90 °C. ^b ^Isolated yields.

We also studied the reactivity of propargyl alcohol (**4b)** with aryl and heteroaryl halides in the presence of 0.1–0.01 mol% of catalyst (Entries 16–28). Generally, the reaction rates for the coupling of aryl and heteroaryl halides with **4b** were slower than those for coupling with **4a**, and reaction of aryl iodides with **4b** at 90 °C gave the desired products in high yields (Entries 16–18). The use of **1d** afforded only 10% of product under the reaction conditions described (Entry 19), while for electron-poor aryl bromides, excellent yields were obtained (Entries 20–23). In the case of **1f,** using a 0.01 mol% catalyst loading, a TON of 9,800 was achieved, which is comparable with the reported efficiency of homogeneous catalysts [17b,f,g] (Entry 22). On the other hand, coupling of **1h** with **4b** gave a yield of only 15% (Entry 24). As for heteroaryl halides, the use of bromothiophenes **1j** and **1k** did not provide the products in good yields (Entries 25 and 26), while with halopyridines, the coupling products were obtained in good to excellent yields (Entries 27 and 28). The coupling of 3-butyn-1-ol (**4c**) with aryl and heteroaryl halides at a catalyst loading of 0.1 mol% was also screened, and the corresponding products were obtained in moderate to high yields (Entries 29–34).

### 2.4. Recycling and leaching studies of NS-MCM-41-Pd in the Sonogashira reaction

One of the purposes of designing this catalyst was to enable catalyst recycling for further use in subsequent reactions. In context, aryl iodides, activated aryl bromides, and several terminal alkynes were examined under optimized reaction conditions, and after completion of the initial cycle, the NS-MCM-41-Pd catalyst was extracted by centrifugation from the reaction mixture, washed successively with THF, H_2_O, and THF, and used for the next run with no regeneration treatment. The results using the recycled catalyst are shown in Table 4. In the case of aryl iodides, we found that the activity of the catalyst was completely retained after two recycled runs, giving an overall TON of between 2,450 and 2,960 (Table 4, Entries 1, 3, and 5). For the activated aryl bromides, the NS-MCM-41-Pd catalyst also exhibited high TONs in the recycled runs (Entries 2 and 4), but a gradual decrease in catalytic activity was observed in the reaction of **1f** with **4b** (Entry 6).

**Table 4:** Sonogashira coupling reaction catalyzed by recycled nanosized MCM-41-Pd.^a^

Entry	Aryl halide	Alkyne	Solvent/Base	T (°C)	t (h)	Yield, %^ b^ (TON)		
						Initial cycle	1st recycle	2nd recycle
1	**1a**	**2a**	Et_3_N/Et_3_N	50	3	99 (990)	99 (990)	98 (980)
2	**1f**	**2a**	NMP/Et_3_N^c^	90	6	98 (980)	93 (930)	91 (910)
3	**1a**	**4a**	Et_3_N/Et_3_N	90	3	94 (940)	90 (900)	88 (880)
4	**1f**	**4a**	Et_3_N/Et_3_N	90	3	98 (980)	99 (990)	95 (950)
5^d^	**1b**	**4b**	Et_3_N/Et_3_N	90	3	83 (830)	82 (820)	80 (800)
6^d^	**1f**	**4b**	Et_3_N/Et_3_N	90	3	98 (980)	88 (880)	76 (760)

^a ^Reaction conditions: [**1**]:[**2** or **4**]:[Pd]:[CuI]:[PPh_3_] = 1,000:1,100:1:2:2. ^b ^Isolated yields. ^c ^3 equiv based on **1** was used as a base. ^d^[**1**]:[**4b**]:[Pd]:[CuI]:[PPh_3_] = 1,000:1,500:1:2:2.

**Figure 2:**
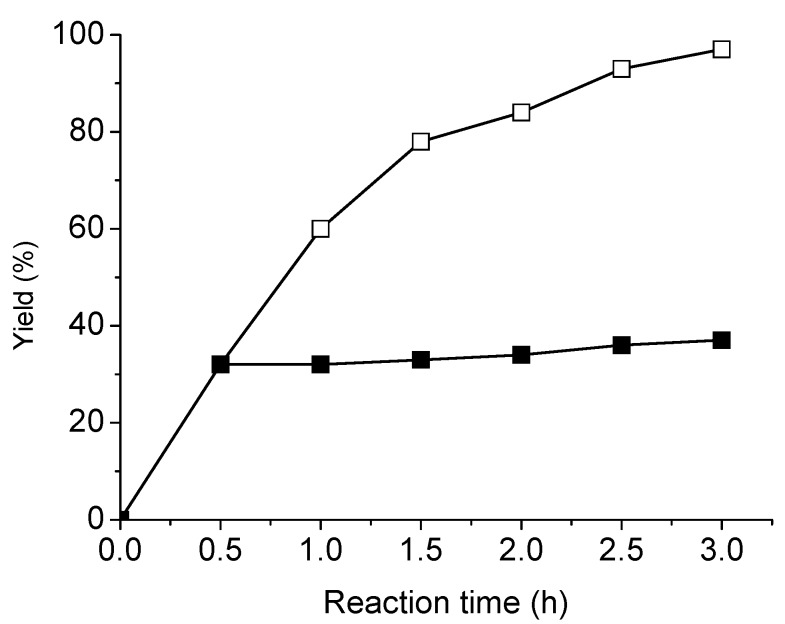
Plot of yield versus time with hot-filtration for 0.5 h of reaction at 50 °C (■) and a comparative reaction without hot-filtration (□). [**1a**]:[**2a**]:[Pd]:[CuI]:[PPh_3_] = 1000/1100/1/2/2.

Several studies have successfully determined the amount of metal leaching using a hot-filtration technique, and this method was therefore used in this study to examine the activity of the catalyst with regards to metal leaching [45,46,47,48,56,57,58]. A reaction mixture of **1a** with **2a** in the above-described catalytic system was stirred at 50 °C for 30 min, resulting in a GC yield of 32%. The hot reaction mixture was then filtered through a dried Celite pad under nitrogen to remove the NS-MCM-41-Pd catalyst and any insoluble species, and the clear filtrate was introduced to another Schlenk tube at 50 °C. Further detection by GC demonstrated improvement of the yield to only 37% after 3 h, even in the presence of additional CuI and PPh_3_ (Figure 2). This result shows that no active species were dissolved in the solution to catalyze the coupling reaction. We further determined the Pd-content in the filtrate by ICP-MASS, and only 0.5 ppm of palladium was found in the solution, which indicated that the catalytic activity may mainly result from the grafted palladium complex. However, another pathway of the Sonogashira reaction is catalysis by a dissolved Pd-species that occurs inside the channels of NS-MCM-41, and this pathway cannot be excluded.

## 3. Experimental

### 3.1. General

All reactions involving air- and moisture- sensitive conditions were carried out under a dry nitrogen atmosphere. *N*-Methylpyrrolidinone (NMP) was distilled under reduced pressure before use; Et_3_N and Bu_3_N were distilled from KOH; and toluene was distilled from sodium benzophenone ketyl. Aryl halides and terminal alkynes were purchased from ARCOS Co. Ltd and were used without further purification. 4,4’-Bis(bromomethyl)-2,2’-bipyridine [59,60], nanosized MCM-41 [61], and NS-MCM-41-Pd [55,56] were prepared according to the previously-published procedures. Melting points were recorded on melting point apparatus and were uncorrected. ^1^H- and ^13^C-NMR spectra were recorded in CDCl_3_ or C_6_D_6_ solution at 25 °C on a Varian 200 NMR spectrometer. GC analysis was performed on an SRI 8610C instrument equipped with a fused silica capillary column.

### 3.2. General procedure for the Sonogashira coupling

Under a nitrogen atmosphere, a mixture of NS-MCM-41-Pd (50 mg, 7.5 × 10^-3^ mmol-Pd), CuI (2.9 mg, 1.5 × 10^-2^ mmol), and PPh_3_ (3.9 mg, 1.5 × 10^-2^ mmol) in Et_3_N (15 mL) in a 50 mL Schlenk tube was charged with aryl halide (7.5 mmol) and terminal alkyne (8.3 mmol; in the case of **4b**, 11.3 mmol was used), and the reaction mixture stirred at 50 °C or 90 °C. After cooling to room temperature, the resulting solution was passed through a short silica gel column with ethyl acetate as the eluent to remove ammonium salt. After evaporation of the solvent, column chromatography on silica gel afforded the desired product.

*Diphenylacetylene* (**3a**). White solid. m.p. 60–61 °C (lit.[47] 60–61 °C). ^1^H-NMR: δ 7.32–7.34 (m, 6H), 7.51–7.56 (m, 4H); ^13^C-NMR: δ 89.2 (2C), 122.9 (2C), 127.8 (4C), 127.9 (2C), 131.2 (4C).

*4-(Phenylethynyl)benzonitrile* (**3b**). Pale yellow solid. m.p. 108–110 °C (lit.[62] 106–108 °C). ^1^H-NMR: δ 7.35–7.38 (m, 3H), 7.51–7.55 (m, 2H), 7.60–7.61 (m, 4H); ^13^C-NMR: δ 87.6, 93.6, 111.1, 118.2, 121.8, 127.8, 128.1 (2C), 128.7, 131.3 (2C), 131.6 (2C), 131.6 (2C).

*4-(Phenylethynyl)anisole* (**3c**). Brown solid. m.p. 60–61 °C (lit.[62] 60.6 °C). ^1^H-NMR: δ 2.88 (s, 3H), 6.28–6.33 (m, 2H), 6.69–6.70 (m, 3H), 7.15–7.20 (m, 4H); ^13^C-NMR: δ 55.4, 87.9 (2C), 113.7 (2C), 115.0, 123.2, 127.5, 127.9 (2C), 131.0 (2C), 132.6 (2C), 158.9.

*4-(Phenylethynyl)acetophenone* (**3d**). Brown solid. m.p. 97–99 °C (lit.[47] 98–99 °C). ^1^H-NMR: δ 2.60 (s, 3H), 7.34–7.37 (m, 3H), 7.51–7.61 (m, 2H), 7.78–7.83 (m, 2H), 7.91–7.95 (m, 2H); ^13^C-NMR: δ 27.0, 88.5, 92.6, 122.3, 127.8, 127.9 (2C), 128.0 (2C), 128.4, 131.3 (2C), 132.0 (2C), 135.7, 196.4.

*4-(Phenylethynyl)nitrobenzene* (**3e**). Yellow solid. m.p. 116–117 °C (lit.[62] 114–116 °C). ^1^H-NMR: δ 7.34–7.39 (m, 3H) 7.52–7.56 (m, 2H), 7.63–7.66 (m, 2H), 8.19–8.22 (m, 2H); ^13^C-NMR: δ 87.4, 94.6, 121.7, 123.3, 128.1 (2C), 128.9 (2C), 129.8, 131.4 (2C), 131.8 (2C), 146.4.

*Phenyl-(4-chlorophenyl)acetylene* (**3f**). White solid. m.p. 82–83 °C (lit.[47] 82–83 °C).^ 1^H-NMR: δ 7.30–7.36 (m, 5H), 7.43–7.46 (m, 2H), 7.51–7.53 (m, 2H); ^13^C-NMR: δ 88.1, 90.2, 121.4, 122.5, 128.0 (2C), 128.1, 128.3 (2C), 131.2 (2C), 132.4 (2C), 133.8.

*2-(Theinylethynyl)benzene* (**3g**). Pale yellow solid. m.p. 50–52 °C (lit.[63] 51–53 °C).^ 1^H-NMR: δ 7.00–7.02 (m, 1H), 7.28–7.29 (m, 2H), 7.34–7.35 (m, 3H), 7.51–7.52 (m, 2H); ^13^C-NMR: δ 82.5, 92.9, 126.7, 126.8, 127.9 (2C), 128.0, 128.1, 131.0 (2C), 131.5, 132.0.

*3-(Theinylethynyl)benzene* (**3h**). Brown solid. m.p. 50–52 °C (lit.[64] 52–54 °C).^ 1^H-NMR: δ 7.20–7.22 (m, 1H), 7.29–7.32 (m, 1H), 7.33–7.37 (m, 3H), 7.52–7.54 (m, 3H); ^13^C-NMR: δ 84.4, 88.8, 121.9, 122.8, 125.0, 127.8, 127.9 (2C), 128.2, 129.4, 131.1 (2C).

*2-(Phenylethynyl)pyridine* (**3i**) [65]. Colorless liquid. ^1^H-NMR: δ 7.15–7.18 (m, 1H), 7.29–7.32 (m, 3H), 7.45–7.47 (m, 1H), 7.54–7.62 (m, 3H), 8.55–8.57 (m, 1H); ^13^C-NMR: δ 88.4, 88.9, 121.7, 122.3, 126.6, 127.9, 128.4, 131.5, 135.6, 142.8, 149.3.

*3-(Phenylethynyl)pyridine* (**3j**). Yellow solid. m.p. 50–51 °C (lit.[66] 50–51 °C). ^1^H-NMR: δ 7.23–7.26 (m, 1H), 7.33–7.36 (m, 3H), 7.52–7.55 (m, 2H), 7.76–7.79 (m, 1H), 8.51–8.53 (m, 1H), 8.75–8.76 (m, 1H); ^13^C-NMR: δ 85.8, 92.4, 121.4, 120.0, 122.0, 122.6, 128.0 (2C), 128.3, 131.2 (2C), 137.8, 147.9, 151.6.

*2-Methyl-4-phenyl-3-butyn-2-ol* (**5a**). Yellow solid. m.p. 53–54 °C (lit.[67] 53.5–54.5 °C). ^1^H-NMR: δ 1.61 (s, 6H), 2.01 (s, 1H), 7.27–7.28 (m, 3H), 7.38–7.41 (m, 2H); ^13^C-NMR: δ 31.8 (2C), 65.7, 82.1, 104.1, 122.3, 127.8 (2C), 127.9, 131.2 (2C).

*2-Methyl-4-(4’-cyano)phenyl-3-butyn-2-ol* (**5b**) [68]. Yellow solid. m.p. 69–70 °C (lit.[69] 68.5–69.5 °C). ^1^H-NMR: δ 1.62 (s, 6H), 2.12 (s, 1H), 7.47 (d, *J* = 6.4 Hz, 2H), 7.57 (d, *J* = 6.4 Hz, 2H); ^13^C-NMR: δ 31.6 (2C), 65.7, 80.6, 98.0, 111.3, 118.1, 127.3, 131.5 (2C), 131.7 (2C).

*2-Methyl-4-(4’-methoxy)phenyl-3-butyn-2-ol* (**5c**) [70]. Yellow oil. ^1^H-NMR: δ 1.59 (s, 6H), 2.24 (s, 1H), 3.76 (s, 3H), 6.79 (d, *J* = 8.0 Hz, 2H), 7.31 (d, *J* = 8.0 Hz, 2H); ^13^C-NMR: δ 31.8 (2C), 55.3, 65.6, 81.8, 92.3, 113.5 (2C), 114.5, 132.5 (2C), 158.7.

*2-Methyl-4-(4’-acetyl)phenyl-3-butyn-2-ol* (**5d**) [70]. Yellow oil. ^1^H-NMR: δ 1.59 (s, 6H), 2.53 (s, 3H), 2.83 (s, 1H), 7.40 (d, *J* = 6.8 Hz, 2H), 7.81 (d, *J* = 6.8 Hz, 2H); ^13^C-NMR: δ 26.9, 31.5 (2C), 65.4, 81.0, 97.1, 127.3, 127.7 (2C), 131.2 (2C), 135.5, 196.6.

*2-Methyl-4-(4’-nitro)phenyl-3-butyn-2-ol* (**5e**). Brown solid. m.p. 100–102 °C (lit.[71] 102 °C). ^1^H-NMR: δ 1.62 (s, 6H), 2.09 (s, 1H), 7.49 (d, *J* = 7.6 Hz, 2H), 8.14 (d, *J* = 7.6 Hz, 2H); ^13^C-NMR: δ 31.5 (2C), 65.7, 80.4, 99.8, 123.1 (2C), 129.3, 131.9 (2C), 146.5.

*2-Methyl-4-(4’-chloro)phenyl-3-butyn-2-ol* (**5f**). White solid. m.p. 55–56 °C (lit.[72] 55–57 °C). ^1^H-NMR: δ 1.60 (s, 6H), 2.11 (s, 1H), 7.25 (d, *J* = 8.4 Hz, 2H), 7.31 (d, *J* = 8.4 Hz, 2H); ^13^C-NMR: δ 31.7 (2C), 65.7, 81.0, 94.5, 120.8, 128.2 (2C), 132.4 (2C), 133.8.

*2-Methyl-4-(2-thienyl)-3-butyn-2-ol* (**5g**). Off-white solid. m.p. 56–57 °C (lit.[73] 54 °C). ^1^H-NMR: δ 1.58 (s, 6H), 2.51 (s, 1H), 6.91–6.93 (m, 1H), 7.14–7.15 (m, 1H), 7.19–7.21 (m, 1H); ^13^C-NMR: δ 31.6 (2C), 65.7, 75.4, 97.3, 122.2, 126.5, 126.6, 131.5.

*2-Methyl-4-(3-thienyl)-3-butyn-2-ol* (**5h**). Brown solid. m.p. 54–56 °C (lit.[74] 56 °C). ^1^H-NMR: δ 1.61 (s, 6H), 2.12 (s, 1H), 7.08–7.09 (m, 1H), 7.24–7.26 (m, 1H), 7.41–7.42 (m, 1H); ^13^C-NMR: δ 31.8 (2C), 65.7, 77.3, 93.2, 124.9, 128.2, 129.4.

*2-Methyl-4-(2-pyridyl)-3-butyn-2-ol* (**5i**). Off-white solid. m.p. 60–61 °C (lit.[25] 61 °C). ^1^H-NMR: δ 1.59 (s, 6H), 2.95 (s, 1H), 7.11–7.15 (m, 1H), 7.29–7.32 (m, 1H), 7.52–7.57 (m, 1H), 8.47–8.49 (m, 1H); ^13^C-NMR: δ 31.4 (2C), 64.9, 80.9, 94.6, 122.4, 126.6, 135.5, 142.3, 148.9.

*2-Methyl-4-(3-pyridyl)-3-butyn-2-ol* (**5j**). Yellow solid. m.p. 55–56 °C (lit.[25] 53 °C). ^1^H-NMR: δ 1.58 (s, 6H), 2.00 (s, 1H), 7.18–7.22 (m, 1H), 7.64–7.67 (m, 1H), 8.44–8.45 (m, 1H), 8.71 (s, 1H); ^13^C-NMR: δ 31.6 (2C), 64.9, 78.1, 98.3, 120.0, 122.8, 138.4, 147.3, 151.3.

*3-Phenyl-2-propyn-1-ol* (**5k**) [75]. Yellow oil. ^1^H-NMR: δ 2.87 (s, 1H), 4.50 (s, 2H), 7.27–7.33 (m, 3H), 7.42–7.45 (m, 2H); ^13^C-NMR: δ 51.4, 85.3, 87.1, 123.1, 127.8 (2C), 127.9, 131.1 (2C).

*3-(4’-Cyano)phenyl-2-propyn-1-ol* (**5l**) [76]. Off-white solid. m.p. 89–91 °C (lit.[77] 87.5–88 °C). ^1^H-NMR: δ 2.01 (s, 1H), 4.50 (s, 2H), 7.48 (d, *J* = 6.4 Hz, 2H), 7.57 (d, *J* = 6.4 Hz, 2H); ^13^C-NMR: δ 51.6, 83.9, 91.6, 111.5, 118.0, 127.1, 131.5 (2C), 131.7 (2C).

*4-(3-Hydroxy-1-propynyl)acetophenone* (**5m**). Yellow solid. m.p. 80–81 °C (lit.[78] 80–81 °C). ^1^H-NMR: δ 2.55 (s, 3H), 2.80 (s, 1H), 4.90 (s, 2H), 7.43 (d, *J* = 8.4 Hz, 2H), 7.82 (d, *J* = 8.4 Hz, 2H); ^13^C-NMR: δ 26.9, 51.5, 84.4, 90.7, 127.1, 127.8 (2C), 131.2 (2C), 135.7, 196.8.

*3-(4’-Nitro)phenyl-2-propyn-1-ol* (**5n**). Yellow solid. m.p. 96–97 °C (lit.[79] 95–96.5 °C). ^1^H-NMR: δ 1.95 (s, 1H), 4.52 (s, 2H), 7.54 (d, *J* = 8.0 Hz, 2H), 8.15 (d, *J* = 8.0 Hz, 2H); ^13^C-NMR: δ 51.6, 83.7, 92.4, 123.2 (2C), 129.0, 131.9 (2C), 146.6.

*3-(4’-Chlorophenyl)-2-propyn-1-ol* (**5o**). Yellow solid. m.p. 77–79 °C (lit.[72] 78.5–79 °C). ^1^H-NMR: δ 2.11 (s, 1H), 4.47 (s, 2H), 7.25 (d, *J* = 8.8 Hz, 2H), 7.33 (d, *J* = 8.8 Hz, 2H); ^13^C-NMR: δ 51.7, 84.5, 88.0, 120.6, 128.2 (2C), 132.4 (2C), 134.1.

*3-(2’-Thiophenyl)-2-propyn-1-ol* (**5p**) [64]. Pale yellow oil. ^1^H-NMR: δ 2.17 (s, 1H), 4.50 (s, 2H), 6.95–6.97 (m, 1H), 7.20–7.21 (m, 1H), 7.25–7.26 (m, 1H); ^13^C-NMR: δ 51.8, 78.8, 91.0, 122.0, 126.6, 127.0, 131.9.

*3-(3’-Thiophenyl)-2-propyn-1-ol* (**5q**) [54]. Brown oil. ^1^H-NMR: δ 3.06 (s, 1H), 4.46 (s, 2H), 7.06–7.08 (m, 1H), 7.19–7.24 (m, 1H), 7.41–7.42 (m, 1H); ^13^C-NMR: δ 51.4, 80.6, 86.8, 121.1, 124.9, 128.6, 129.3.

*3-(2-Pyridyl)-2-propyn-1-ol* (**5r**). White solid. m.p. 83–84 °C (lit.[80] 82 °C). ^1^H-NMR: δ 2.43 (s, 1H), 4.53 (s, 2H), 7.19–7.22 (m, 1H), 7.39–7.41 (m, 1H), 7.60–7.64 (m, 1H), 8.50–8.51 (m, 1H); ^13^C-NMR: δ 51.3, 84.1, 88.3, 122.7, 126.8, 136.0, 142.2, 149.2.

*3-(3-Pyridyl)-2-propyn-1-ol* (**5s**). White solid. m.p. 101–102 °C (lit.[81] 99–100 °C). ^1^H-NMR: δ 2.08 (s, 1H), 4.49 (s, 2H), 7.23–7.36 (m, 1H), 7.70–7.74 (m, 1H), 8.47–8.48 (m, 1H), 8.74–8.75 (m, 1H); ^13^C-NMR: δ 51.0, 81.3, 92.0, 119.9, 122.9, 138.5, 147.6, 151.4.

*4-Phenyl-3-butyn-1-ol* (**5t**) [82]. Light brown oil. ^1^H-NMR: δ 1.84 (t, *J* = 6.2 Hz, 1H), 2.70 (t, *J* = 6.2 Hz, 2H), 3.82 (q, *J* = 6.2 Hz, 2H), 7.26–7.32 (m, 3H), 7.39–7.44 (m, 2H); ^13^C-NMR: δ 23.5, 60.9, 82.1, 86.5, 123.3, 127.7, 128.1 (2C), 131.5 (2C).

*4-(4’-Methoxy)phenyl-3-butyn-1-ol* (**5u**). Pale yellow solid. m.p. 58–59 °C (lit.[83] 61 °C). ^1^H-NMR: δ 1.90 (br, 1H), 2.65 (t, *J* = 6.1 Hz, 2H), 3.77 (t, *J* = 6.1 Hz, 2H), 6.80 (d, *J* = 8.6 Hz, 2H), 7.32 (d, *J* = 8.6 Hz, 2H); ^13^C-NMR: δ 23.6, 55.1, 61.1, 81.9, 84.8, 113.7 (2C), 115.4, 132.8 (2C), 159.1.

*4-(4’-Cyano)phenyl-3-butyn-1-ol* (**5v**) [53]. Pale yellow solid. m.p. 80–81 °C. ^1^H-NMR: δ 1.97 (br, 1H), 2.69 (t, *J* = 6.2 Hz, 2H), 3.81 (t, *J* = 6.2 Hz, 2H), 7.44 (d, *J* = 8.4 Hz, 2H), 7.54 (d, *J* = 8.4 Hz, 2H); ^13^C-NMR: δ 23.6, 60.6, 80.6, 91.8, 110.8, 118.3, 128.4, 131.7 (2C), 132.0 (2C).

*4-(4-Hydroxy-1-butynyl)acetophenone* (**5w**). Pale yellow solid. m.p. 75–77 °C (lit.[53] 74–76 °C). ^1^H-NMR: δ 2.00 (br, 1H), 2.56 (s, 3H), 2.69 (t, *J* = 6.2 Hz, 2H), 3.81 (t, *J* = 6.2 Hz, 2H), 7.45 (d, *J* = 8.2 Hz, 2H), 7.85 (d, *J* = 8.2 Hz, 2H); ^13^C-NMR: δ 23.7, 26.4, 60.8, 81.4, 90.5, 128.0 (2C), 128.4, 131.6 (2C), 135.7, 197.4.

*4-(2-Thiophenyl)-3-butyn-1-ol* (**5x**) [54]. Light brown oil. ^1^H-NMR: δ 1.93 (br, 1H), 2.69 (t, *J* = 6.2 Hz, 2H), 3.79 (t, *J* = 6.2 Hz, 2H), 6.92 (dd, *J* = 5.1, 3.6 Hz, 1H), 7.14 (d, *J* = 3.6 Hz, 1H), 7.18 (d, *J* = 5.2 Hz, 1H); ^13^C-NMR: δ 24.0, 60.9, 75.4, 90.5, 123.3, 126.2, 126.7, 131.4.

*4-(2-Pyridyl)-3-butyn-1-ol* (**5y**) [25]. Light brown oil. ^1^H NMR: δ 2.72 (t, *J* = 6.0 Hz, 2H), 3.06 (br, 1H), 3.87 (t, *J* = 6.0 Hz, 2H), 7.18–7.25 (m, 1H), 7.39 (d, *J* = 7.8 Hz, 1H), 7.64 (td, *J* = 7.8, 2.0 Hz, 1H), 8.54 (d, *J* = 5.0 Hz, 1H); ^13^C-NMR: δ 23.3, 59.9, 80.7, 88.4, 122.2, 126.4, 136.0, 142.7, 148.8.

### 3.3. General procedure for recycling of nanosized MCM-41-Pd

Under a nitrogen atmosphere, a 50 mL Schlenk tube was charged with NS-MCM-41-Pd (50 mg, 7.5 × 10^-3^ mmol-Pd), CuI (2.9 mg, 1.5 × 10^-2^ mmol), PPh_3_ (3.9 mg, 1.5 × 10^-2^ mmol), Et_3_N (15 mL), aryl halide (7.5 mmol), and terminal alkyne (8.3 mmol; in the case of **4b**, 11.3 mmol was used). The mixture was stirred at 50 °C for 3 h (6 h for Entry 2) and then cooled to room temperature. Recovery of NS-MCM-41-Pd was achieved by centrifugation and successive washes with THF, H_2_O, and THF (2 × 40 mL each washing). The solid was then dried under vacuum overnight and used for the next run.

## 4. Conclusions

In conclusion, NS-MCM-41-Pd is a highly efficient and recyclable catalyst for the coupling of a wide variety of aryl and heteroaryl halides with terminal alkynes, requiring catalyst loadings as low as 0.01 mol% for a single run. The NS-MCM-41-Pd catalyst also exhibited excellent reusability when a catalyst loading of only 0.1 mol% was employed for the recycling studies. The results of this study demonstrate the usefulness of anchored palladium bipyridyl complex on mesoporous silica as a heterogeneous catalyst in cross-coupling reactions.
